# Direct Monitoring Reveals Initiation of Turbidity Currents From Extremely Dilute River Plumes

**DOI:** 10.1029/2019GL084526

**Published:** 2019-10-29

**Authors:** Sophie Hage, Matthieu J.B. Cartigny, Esther J. Sumner, Michael A. Clare, John E. Hughes Clarke, Peter J. Talling, D. Gwyn Lintern, Stephen M. Simmons, Ricardo Silva Jacinto, Age J. Vellinga, Joshua R. Allin, Maria Azpiroz‐Zabala, Jenny A. Gales, Jamie L. Hizzett, James E. Hunt, Alessandro Mozzato, Daniel R. Parsons, Ed L. Pope, Cooper D. Stacey, William O. Symons, Mark E. Vardy, Camilla Watts

**Affiliations:** ^1^ National Oceanography Centre Southampton Southampton UK; ^2^ School of Ocean and Earth Sciences University of Southampton Southampton UK; ^3^ Department of Geography Durham University Durham UK; ^4^ Center for Coastal and Ocean Mapping University of New Hampshire Durham NH USA; ^5^ Geological Survey of Canada Natural Resources Canada Sidney British Columbia Canada; ^6^ Energy and Environment Institute University of Hull Hull UK; ^7^ Marine Geosciences Unit IFREMER Centre de Brest Plouzané France; ^8^ Geotek Ltd Daventry UK; ^9^ Faculty of Civil Engineering and Geosciences Delft University of Technology Delft The Netherlands; ^10^ School of Biological and Marine Sciences University of Plymouth Plymouth UK; ^11^ CGG Robertson North Wales UK

## Abstract

Rivers (on land) and turbidity currents (in the ocean) are the most important sediment transport processes on Earth. Yet how rivers generate turbidity currents as they enter the coastal ocean remains poorly understood. The current paradigm, based on laboratory experiments, is that turbidity currents are triggered when river plumes exceed a threshold sediment concentration of ~1 kg/m^3^. Here we present direct observations of an exceptionally dilute river plume, with sediment concentrations 1 order of magnitude below this threshold (0.07 kg/m^3^), which generated a fast (1.5 m/s), erosive, short‐lived (6 min) turbidity current. However, no turbidity current occurred during subsequent river plumes. We infer that turbidity currents are generated when fine sediment, accumulating in a tidal turbidity maximum, is released during spring tide. This means that very dilute river plumes can generate turbidity currents more frequently and in a wider range of locations than previously thought.

## Key Points

- Here we document for the first time how very dilute (up to 0.07 kg/m^3^) river plumes can generate powerful turbidity currents
- Such low sediment concentrations are 20 times lower than those predicted by past theory and experiments
- Therefore, turbidity currents are likely to be much more frequent and occur at a far wider range of locations than previously thought

## 1 Introduction

Turbidity currents are seafloor‐hugging flows that are driven by their suspended sediment (Daly, 1936; Middleton & Hampton, 1973). These flows are the main process transporting terrestrial sediment from river mouths into the deep sea. The combination of rivers and turbidity currents accounts for the majority of global sediment transport (Talling, 2014). However, the link between rivers and turbidity currents is poorly understood because there are few direct measurements of how turbidity currents are generated at river mouths (e.g., Ayranci et al., 2012; Hizzett et al., 2018). Understanding this link is important for understanding the global redistribution of sediment, organic matter (Liu et al., 2012), and pollutants such as plastic (Kane & Clare, 2019).

**Figure 1:**
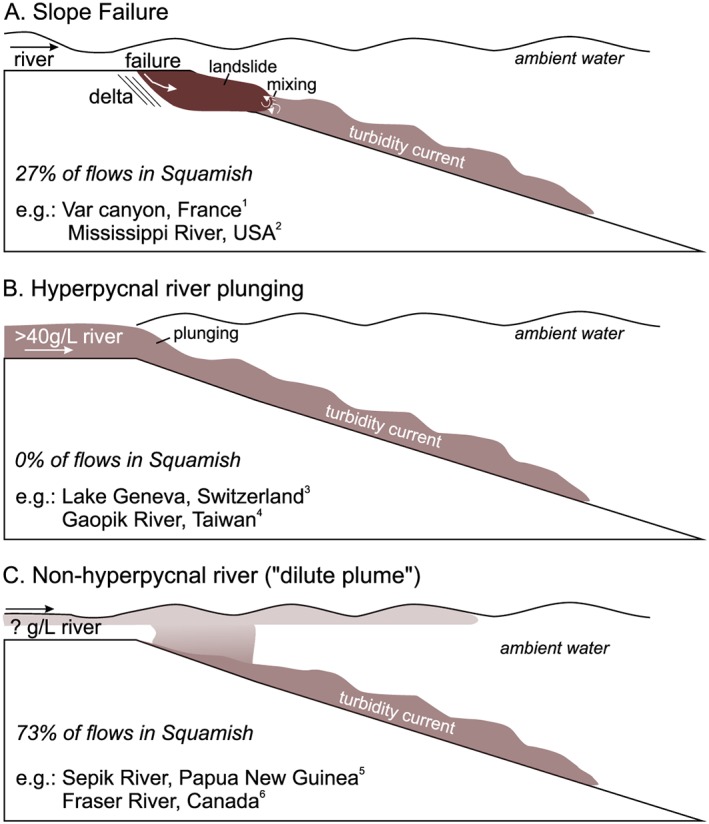
(a–c) Mechanisms triggering turbidity currents at river mouths proposed in the literature. Percentage of flows triggered in Squamish by each mechanism are based on Hizzett et al. (2018). References for given examples are 1: Piper & Savoye, 1993; Mulder et al., 1997. 2: Obelcz et al., 2017. 3: Girardclos et al., 2012. 4: Carter et al., 2012; Liu et al., 2012. 5: Kineke et al., 1995. 6: Lintern et al., 2016.

Three main processes have been proposed for the initiation of turbidity currents from river plumes (Clare et al., 2016; Piper & Normark, 2009). First, delta slope failures generate submarine landslides that evolve into turbidity currents (Figure 1a; Piper & Savoye, 1993; Clare et al., 2016; Obelcz et al., 2017). Second, river plumes that are denser than seawater (>40 kg/m^3^ of sediment), directly feed turbidity currents (Figure 1b; Mulder & Syvistski, 1995; Liu et al., 2012); this is commonly called a plunging hyperpycnal flow. Only 9 out of 150 rivers studied by Mulder and Syvitski (1995) have sufficient concentrations to enable plunging hyperpycnal flow. Third, experiments suggest that turbidity currents are generated by dilute river plumes with sediment concentrations as low as 1 kg/m^3^ (Figure 1c; Parsons et al., 2001) if the plume locally becomes denser than ambient seawater (by double diffusion or settling‐driven convection; Hoyal et al., 1999a, 1999b; Jazi & Wells, 2016; Parsons et al., 2001; Sutherland et al., 2018). This 1‐kg/m^3^ threshold implies that 61 of the 150 studied rivers studied by Mulder and Syvitski (1995) can generate turbidity currents.

In this paper, we define that a river plume has initiated a turbidity current once the flow can erode the seabed. A small number of field studies have suggested that rivers with suspended sediment concentrations less than the 1‐kg/m^3^ threshold can generate turbidity currents. For example, turbidity currents were reported offshore from the Sepik River (sediment concentrations 0.04 to 0.25 kg/m^3^; Kineke et al., 1995) and the Fraser River (sediment concentrations 0.18 kg/m^3^; Ayranci et al., 2012; Lintern et al., 2016). This implies that there could be a fourth mechanism for generating turbidity currents at river mouths. Importantly, such very dilute sediment concentrations are reached by 144 of the 150 rivers studied by Mulder and Syvitski (1995), implying that almost all rivers may directly initiate turbidity currents.

The physical process (es) that generate turbidity currents from very dilute river plumes are not yet understood due to an absence of real‐world observations. Here we present the first observations of how a turbidity current is generated by a dilute river plume. This was achieved by deploying an array of sensors from both stationary and moving vessels at a fjord‐head delta.

Our first aim is to understand how very dilute rivers generate turbidity currents. We document the evolution of a dilute river plume throughout multiple tidal cycles. We propose a new mechanism that explains the formation of a turbidity current from this plume. Our second aim is to understand the implications of this new mechanism for turbidity current triggering globally.

## 2 Study Site

**Figure 2:**
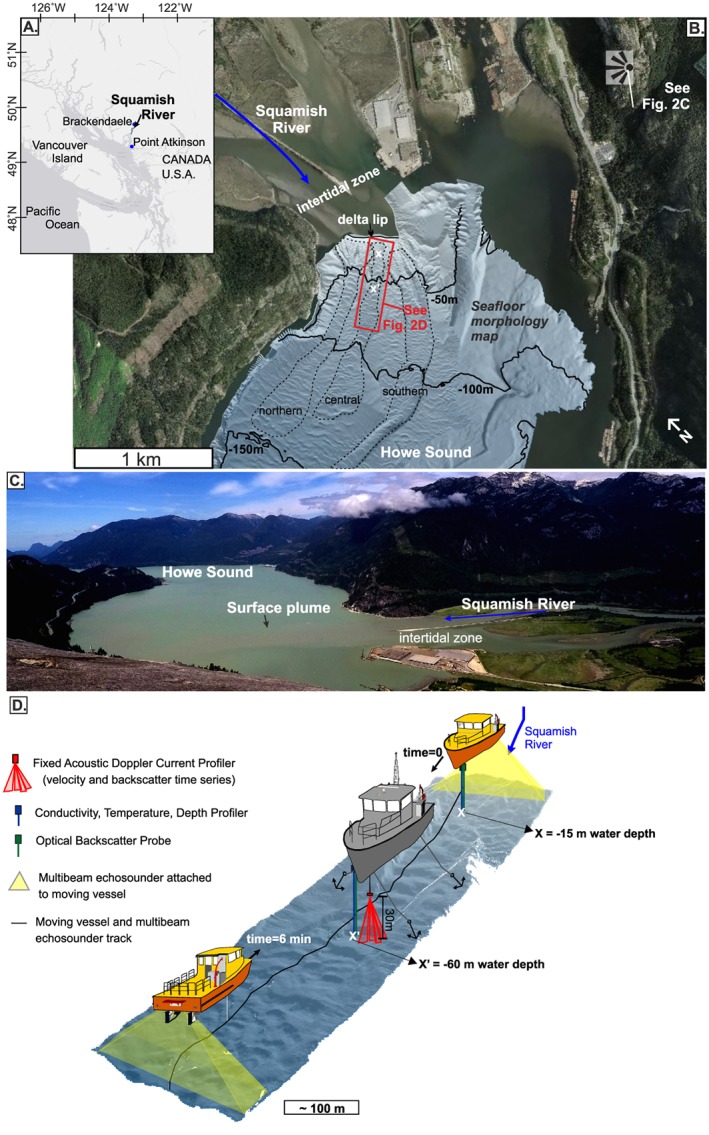
(a–d) Setting and field deployment. (a) Location of Squamish in British Columbia (Canada). (b) Squamish River entering Howe Sound Fjord and bathymetric map of the seafloor. (c) Photograph showing the Squamish River and its plume entering Howe Sound fjord. (d) Three‐dimensional view of the instrument setup in the central submarine channel. X and X' are the locations shown in Figure 4.

The Squamish Delta lies at the mouth of the Squamish River in Howe Sound, a fjord in British Columbia, Canada (Figure 2a). This fjord has a shallow surface layer (~2 m) comprising turbid fresh water derived from the Squamish River, underlain by saline marine water (Syvitski & Murray, 1981). Tides in Howe Sound are mixed semidiurnal with a macrotidal range of ~5 m (Buckley, 1977).

Three sandy submarine channels lie downstream of the delta lip. These channels have been mapped repeatedly since 2011 (Figure 2b, Hughes Clarke et al., 2012, 2014; Hughes Clarke, 2016; Hage et al., 2018), and several turbidity currents have been monitored (typical velocities are 0.5–3 m/s; Hughes Clarke, 2016). These turbidity currents are erosional because they cause movement of upstream‐migrating bedforms within the channels (Hughes Clarke, 2016). Turbidity currents predominantly occur at low tide and when the river discharge exceeds 250 m^3^/s (Clare et al., 2016). In 2011, 106 turbidity currents were monitored: 27% of flows were triggered by slope failures on the delta lip, and 73% of flows were associated with dilute plumes (Hizzett et al., 2018). The Squamish River does not reach the sediment concentrations (~40 kg/m^3^) needed for wholescale plunging (Mulder & Syvitski, 1995) or the 1‐kg/m^3^ threshold to undergo double diffusion settling (Parsons et al., 2001). The Squamish Delta is thus an ideal location to measure how very dilute river plumes generate turbidity currents.

**Figure 3:**
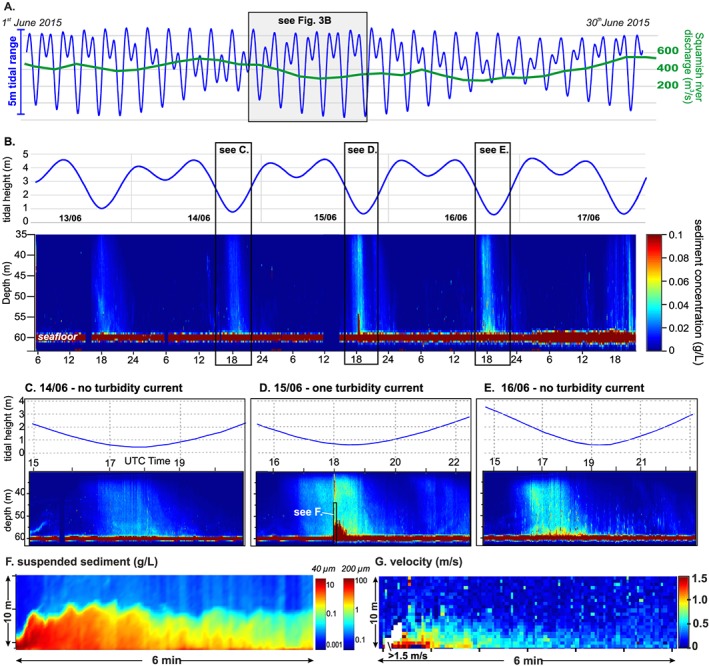
Acoustic Doppler current profiler results. (a) Tides observed at Atkinson Station and Squamish River discharge measured at Brackendaele in June 2015. (b) Tides and suspended sediment time series at fixed vessel location (Figure 2) from 13 to 17 June 2015. Suspended sediment was obtained after inversion of a 600‐kHz Acoustic Doppler current profiler backscatter (assuming grain size of 40 μm or a grain size of 200 μm). (c) Tide and suspended sediment time series on 14 June. (d) Tide and suspended sediment times series on 16 June. (e) Tide and suspended sediment time series on 15 June. (f) Suspended sediment in the turbidity current (assuming grain size distribution with D50 = 200 μm). (g) Velocity magnitude of the turbidity current. Note: These time series images cover 35 to 60 m of water depth, and thus these only show the lower layer imaged in Figure 5a.

We collected observations from 13–17 June 2015 in the central submarine channel (Figures 2b and 2d). River discharge was low (300–400 m^3^/s) for summertime, but higher than the minimum discharge associated with turbidity current generation. Our observations encompassed several tidal cycles, when the tidal amplitude (3.5 to 4 m) was building toward spring tide (Figure 3a).

## 3 Methods

We deployed instruments from two research vessels for 5 days (Figure 2d). The first vessel (RV Strickland) was moored above the central channel, 300 m downstream of the delta lip, at a water depth of 60 m. This stationary vessel was used to suspend a down‐looking 600‐kHz acoustic Doppler current profiler (ADCP, Figure 3) 30 m above seafloor to detect turbidity currents and measure their velocity and collect suspended sediment samples from the water column to calibrate our acoustic measurements. The second vessel (RV Heron) repeatedly surveyed the central channel every 12 min, for a 3‐hr period around low tide. This moving vessel carried two multibeam echosounders, an optical backscatter probe (OBS) and conductivity, temperature, depth probe (CTD) that were raised and lowered to profile the water column.

### 3.1 Velocity and Concentration Measurements

The ADCP was used to measure (1) velocity and (2) acoustic backscatter of the plume and turbidity current, which was then inverted to suspended sediment concentration using established methods (e.g., Azpiroz‐Zabala et al., 2017; Downing et al., 1995; Thorne & Hurther, 2014). Backscatter was corrected for water attenuation and spherical spreading of the acoustic waves (Downing et al., 1995). Corrected backscatter was then inverted with sediment concentration of the flow, assuming a uniform grain‐size distribution (40‐μm D50 in the plume; 200‐μm D50 in the turbidity current—based on sediment samples collected in the water column). There is good agreement (±0.005 kg/m^3^) between the concentration calculated from the inversion and the measurements from sediment sampling (supporting information Figure S7).

### 3.2 Salinity, Temperature, and Suspended Sediment Concentrations

CTD and OBS probes were deployed from the moving vessel at two locations in the central channel. The proximal location was 100 m from the delta lip, at 15‐m water depth. The distal (background) location was 500 m from the delta lip, in 60‐m water depth (Figure 2d). CTD profiles enabled derivation of ambient water density (Figure 5a). OBS probe voltages were converted to sediment concentration by calibration with suspended sediment samples (supporting information Figure S8). Salinity, temperature, and suspended sediment concentrations were combined to derive the density profiles at the proximal and distal locations in the river plume.

**Figure 4:**
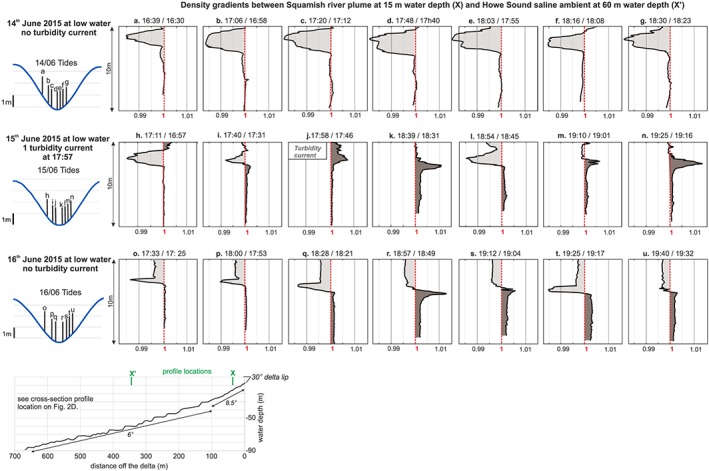
(a–u) Gradient profiles between water density 100 m off the Squamish Delta lip (i.e., 15‐m water depth) and 500 m off the Delta lip (i.e., 60‐m water depth). Water density is based on salinity, temperature (measured by the conductivity, temperature, depth profiler), and suspended sediment concentrations (obtained after calibration of the optical backscatter probe). Profile locations correspond to the two locations shown in Figure 1d. Density gradients <1 (light brown) correspond to conditions where the river plume is lighter than the saline ambient (i.e., added river sediment is not able to overcome the saline water). Density gradients >1 (dark brown) corresponds to conditions where the river plume is heavier than saline ambient due to mixing between riverine sediment and salt.

We computed horizontal density gradients within the top 10 m of the water column by comparing density values at the same water depth within the river plume (proximal location) and the ambient saline background (distal location, Figure 4). Density gradients <1 correspond to the river plume being lighter than the saline background water, implying that the sediment‐laden water is confined against the delta by the salt water. Density gradients >1 correspond to the river plume being denser than the saline background water, such that the sediment‐laden water can migrate offshore.

### 3.3 Echosounder Profiles

**Figure 5:**
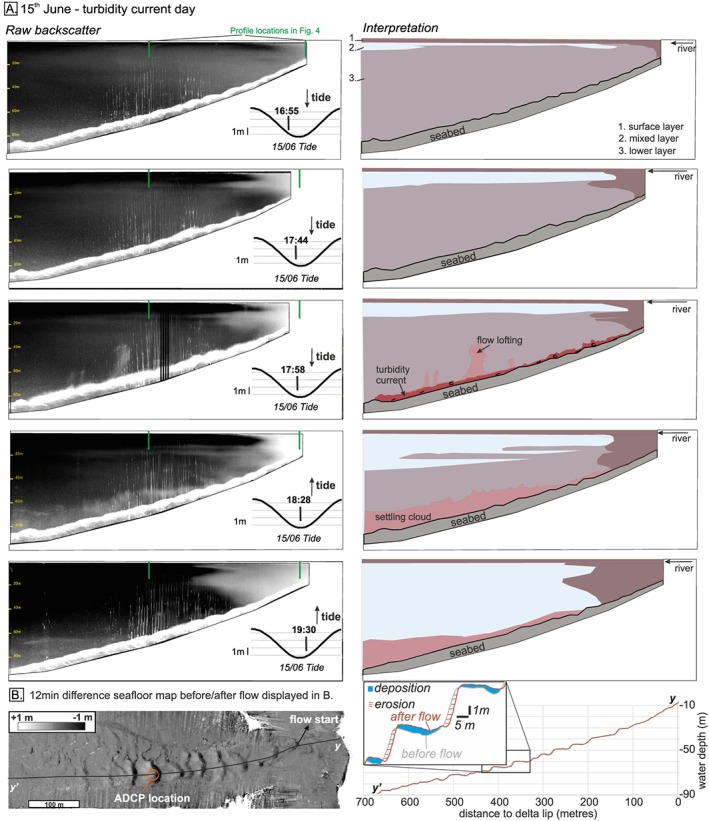
(a) Left column: Five water column transects imaged by a 500‐kHz M3 echosounder on 15 June 2015 along profile track shown in Figure 2c. Right column: Interpretation and transects timing according to tides. (b) Difference map between seafloor morphology 12 min before/after the turbidity current. The turbidity current caused up to 2 m of erosion and up to 1 m of deposition.

A 70 to 100‐kHz multibeam echosounder attached to the moving vessel mapped the seafloor and detected erosion/deposition caused by turbidity currents. A 500‐kHz multibeam echosounder also attached to the moving vessel imaged the suspended sediment (expressed as higher, white backscatter in Figure 5) within the water column from the delta lip to 800 m offshore.

## 4 Results

### 4.1 Water Column Structure and Horizontal Density Gradients

**Figure 6:**
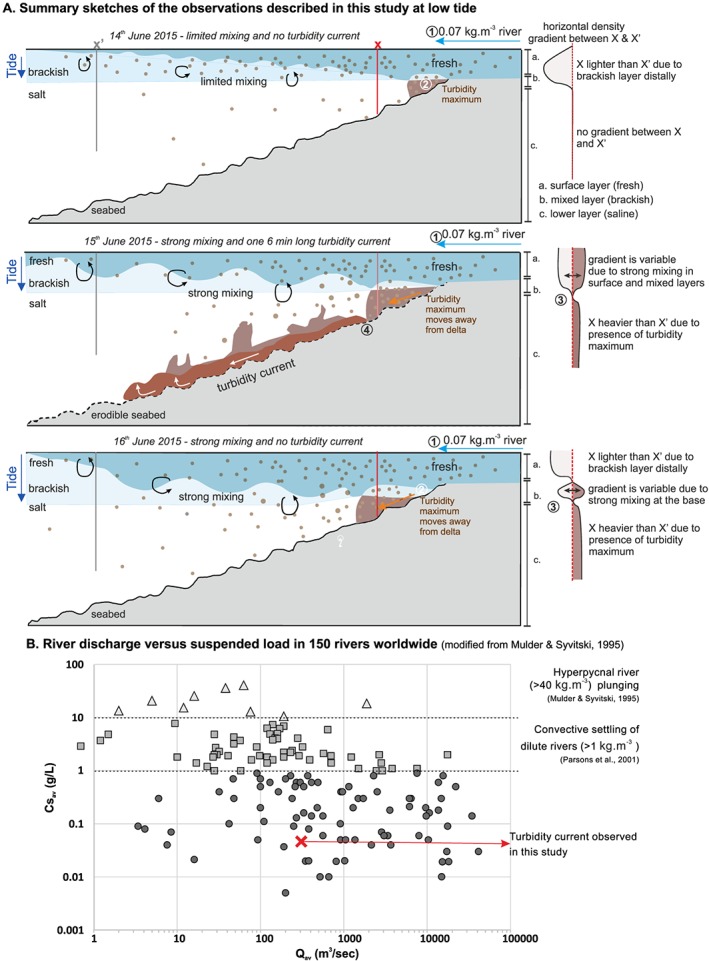
(a) Summary sketches of the observations described in this study. Density ratio sketches correspond to the density difference at the proximal location X compared to the distal location X'. One turbidity current occurred on 15 June in the following steps: 1. river creates a dilute plume at the fjord surface; 2. higher sediment concentration occurs at X in the lower layer due to downslope movement of the turbidity maximum; 3. higher sediment concentration at X generates a positive density gradient, triggering the lower layer to move away from the delta; 4. if the sediment cloud in the lower layer moves away from the delta on an erodible substrate, it can erode and accelerate into a turbidity current. (b) River discharge versus suspended load in 150 rivers worldwide (based on Mulder & Syvitski, 1995), with corresponding mechanisms described in previous studies and in this study.

We divide the water column into three layers (Figures 5a and 6a): (1) The surface layer is ~2 m thick, water is fresh (0–5 practical salinity unit [PSU]), temperature is variable (10–15 °C), and suspended sediment concentrations are high (0.04 to 0.05 kg/m^3^); (2) the mixed layer is from 2 to 5 m in the water column; salinity and temperature increase to ~30 PSU and14 °C, respectively, and suspended sediment decreases to ~0.02 kg/m^3^; (3) the lower layer extends to the seabed, water is saline (29–30 PSU), temperature is 11 to 12 °C, and suspended sediment concentrations are low (0.01 to 0.02 kg/m^3^).

Here we describe horizontal density gradients in each of the three layers. (Figures 2d, 4, and 6a). The surface layer had a density gradient <1 during our study period, as the distal brackish water was always denser than the proximal freshwater in the river plume (Figure 4). The mixed layer had density gradients fluctuating from <1 to >1 on 15 and 16 June, due to strong mixing between salt water and the river plume. The lower layer had neutral density gradients on 14 June, with density gradients in excess of 1 for about 2 hr at low tide on 15 and 16 June. Density gradients >1 are due to enhanced sediment concentrations in the saline lower layer close to the delta.

Importantly, although the >1 density gradient in the lower layer occurs for several hours at low tide on 15 and 16 June, only one 6‐min‐long turbidity current was triggered on 15 June at 17:58 UTM, when the density gradient first exceeded 1 (Figure 4).

### 4.2 Turbidity Current Observations

The turbidity current (peak internal velocity = 1.5 m/s) lasted 6 min, was up to 6 m thick, and was confined within the 10‐m‐deep channel (Figure 3f). Sequential seafloor surveys 12 min before and after the turbidity current (Figure 5b) demonstrate that seafloor erosion began ~100 m downstream of the river mouth thus excluding delta slope failure. These surveys reveal that the turbidity current was most erosive ~500 m downstream of the river mouth. Sediment‐laden water samples from the top of the turbidity current 2 min after the flow began have concentrations of at least 40 kg/m^3^, which is corroborated by the ADCP backscatter data (Figure 3f). The total volume of sediment carried by the turbidity current is estimated to be less than ~670 m^3^ from sequential seafloor surveys and more than 180 m^3^ from the acoustic inversion (which excludes the bottom meter of the flow; Table S2).

### 4.3 Summary

Our results show that sediment settling from a very dilute (~0.07 kg/m^3^) river plume generated a turbidity current that self‐accelerated over a distance of 500 m and became >200 times denser than the initial river plume. Importantly, this turbidity current initiated from a plume that was an order of magnitude is less concentrated than previously thought possible (Parsons et al., 2001); however, subsequent plumes with similar sediment concentrations did not trigger turbidity currents.

## 5 Discussion

We compare our observations with previously suggested trigger mechanisms and threshold plume concentrations and consider when and how dilute river plumes generate turbidity currents. We then discuss the wider implications of our work for the global frequency of turbidity currents offshore from rivers.

### 5.1 A Reduced Threshold Sediment Concentration for Generating Turbidity Currents

Experiments have shown that dilute (0.5–7 kg/m^3^) river plumes entering saline water can settle toward the seabed by double diffusion or settling‐driven convection (Hoyal et al., 1999a, 1999b; Jazi & Wells, 2016; Parsons et al., 2001; Sutherland et al., 2018). In these experiments turbidity currents were only generated when settling plumes had concentrations >1 kg/m^3^ (Parsons et al., 2001). At Squamish Delta, we show that the sediment concentration threshold needed for sediment to reach the *lower layer* and to trigger a turbidity current can be much lower (~0.07 kg/m^3^) than in these previous experimental models (>1 kg/m^3^; Parsons et al., 2001).

However, our study shows that we should not simply consider a fixed river plume sediment concentration threshold, which is because a series of other environmental factors are involved in the generation of turbidity currents by rivers. Below, we discuss a new mechanism that explains how dilute river plumes generate turbidity currents.

### 5.2 How Do Dilute River Plumes Generate Turbidity Currents?

Turbidity currents have been generated by the Squamish River plume during heightened river discharge (>250 m^3^/s) and at low tide (preferentially spring tides; Clare et al., 2016). Here we discuss the role of these two processes in turbidity current generation. Our results reveal that sediment concentrations are highest in the saline lower layer at low water during spring tides (Figure 6a). Locally, increased levels of sediment concentration in tidal deltas occur at the interface between the fresh river water and the saline fjord water; this is called the *turbidity maximum* (Dyer, 1997). Sediment accumulates in this area by the combination of offshore river transport and onshore sediment transport by saline underflow. Where the fresh and salt water meet, they mix and are advected upwards into the mixing layer and away from the delta. The lower velocities in this mixing zone allow sediment accumulation, forming the turbidity maximum; this is often associated with the formation of fine sediment or fluid mud layer on the seafloor (Allen et al., 1980). Increased river discharge and low tide conditions result in faster flows at the river mouth as more water has to flow through a shallower channel. The higher velocities of the river water forces the turbidity maximum away from the delta lip and onto the steeper part of the delta. The ADCP backscatter data show that increased tidal amplitude results in earlier arrival and a higher concentration turbidity maximum (Figure 3). The turbidity maximum on 15 June was sufficiently concentrated to produce the first positive density gradient (Figure 4) in this spring‐neap tidal cycle and thus triggered a turbidity current.

Despite sufficiently concentrated turbidity maxima at the same location on 15 and 16 June, no further turbidity currents were generated. An explanation is that episodic remobilization of seafloor sediment is also needed to trigger (and maintain) a turbidity current. We thus propose that a layer of fine and mobile sediment is deposited on the delta front during the neap part of a tidal cycle. The first turbidity current removes this sediment, and as a result, no further turbidity currents are generated. Unconsolidated seafloor sediments have been observed in other active submarine channels (Curran et al., 2002; Lintern et al., 2016; Paull et al., 2018).

### 5.3 Global Implications for More Frequent and Widespread Turbidity Currents

The major implication of our study is that almost all (144 of 150) rivers in the global database of Mulder and Syvitski (1995) may be able to generate turbidity currents. There may therefore be many settings in which turbidity maxima‐generated turbidity currents occur. However, because we also show that turbidity current generation is not determined by a simple sediment threshold, there is a need for further research in different locations that considers factors such as river discharge, tidal range, seabed gradient, and sediment settling rates.

More frequent generation of turbidity currents at a wider range of locations globally has important implications. Turbidity currents offshore from river mouths often carry large amounts of organic carbon (Liu et al., 2012). This new mechanism for turbidity current generation will increase the dispersal and burial of terrestrial organic carbon in the deep sea. Our work also has implications for how turbidity currents form thick deltaic deposits within the geological record (Hage et al., 2018), as this new triggering mechanism is likely to have been important during sea level lowstand conditions, when more of the world's rivers flowed directly onto the continental slope.

## 6 Conclusion

It was previously thought that rivers needed to exceed a sediment concentration threshold to generate turbidity currents offshore river mouths (e.g., 40 kg/m^3^, Mulder & Syvitski, 1995; 1 kg/m^3^, Parsons et al., 2001). Here we show that rivers with far lower sediment concentrations (0.07 kg/m^3^) can produce local turbidity maxima sufficiently dense to generate powerful turbidity currents. However, these turbidity currents only occur when fine sediment that settled from the dilute plume during lower tidal amplitudes or reduced river discharges is available on the seafloor to be remobilized. Our findings are important as they imply that a far wider range of rivers than previously thought have the potential to generate turbidity currents because there is no fixed sediment threshold that must be exceeded. Understanding the mechanisms that initiate turbidity currents offshore river mouths is crucial because this is the starting point for delivery of terrestrial particles (e.g., organic carbon and microplastics) to the deep sea.

## Supporting information



Supporting Information S1
