# Supplementary material for: Direct Monitoring Reveals Initiation of Turbidity Currents From Extremely Dilute River Plumes
Source: Geophys Res Lett. 2019 Oct 29;46(20):11310–20. doi: 10.1029/2019GL084526 (PMC6919390; doi:10.1029/2019GL084526)
Supplement: Supplementary file 1 — Supporting Information S1 [file GRL-46-11310-s001.docx]

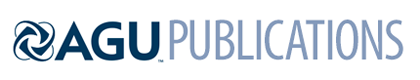


*Geophysical Research Letters*

Supporting Information for

**Direct monitoring reveals initiation of turbidity currents from extremely dilute river plumes**

Sophie Hage^1,2^, Matthieu J.B. Cartigny^3^, Esther J. Sumner^1^, Michael A. Clare^2^, John E. Hughes Clarke^4^, Peter J. Talling^3^, D. Gwyn Lintern^5^, Stephen M. Simmons^6^, Ricardo Silva Jacinto^7^, Age J. Vellinga^1^, Joshua R. Allin^8^, Maria Azpiroz-Zabala^9^, Jenny A. Gales^10^, Jamie L. Hizzett^1^, James E. Hunt^2^, Alessandro Mozzato^1^, Daniel R. Parsons^6^, Ed L. Pope^3^, Cooper D. Stacey^5^, William O. Symons^11^, Mark E. Vardy^2^, Camilla Watts^1^

^1^School of Ocean and Earth Sciences, University of Southampton, European Way Southampton SO14 3ZH, U.K. ^2^National Oceanography Centre Southampton, European Way Southampton SO14 3ZH, U.K. ^3^Department of Geography, Durham University, South Road Durham DH1 3LE, U. K. ^4^ Center for Coastal and Ocean Mapping, University of New Hampshire, Durham, NH, USA, ^5^Natural Resources Canada, Geological Survey of Canada, 9860 W Saanich Road V8L 4B2, Sidney, BC, Canada ^6^Department of Geography, Environment and Earth Sciences, University of Hull, HU6 7RX, U.K. ^7^Marine Geosciences Unit, IFREMER, Centre de Brest, CS10070, 29280 Plouzané, France ^8^ Geotek Long Buckby, England, UK ^9^ Faculty of Civil Engineering and Geosciences, 2628 CN Delft University, The Netherlands ^10^ School of Biological and Marine Sciences, Drake Circus, University of Plymouth, PL48AA,U.K ^11^ CGG Robertson, Llandudno, North Wales, LL30 1SA, UK

Corresponding author: Sophie Hage (Sophie.hage@soton.ac.uk)

**Contents of this file**

Figures S7 to S12

Tables S1, S2


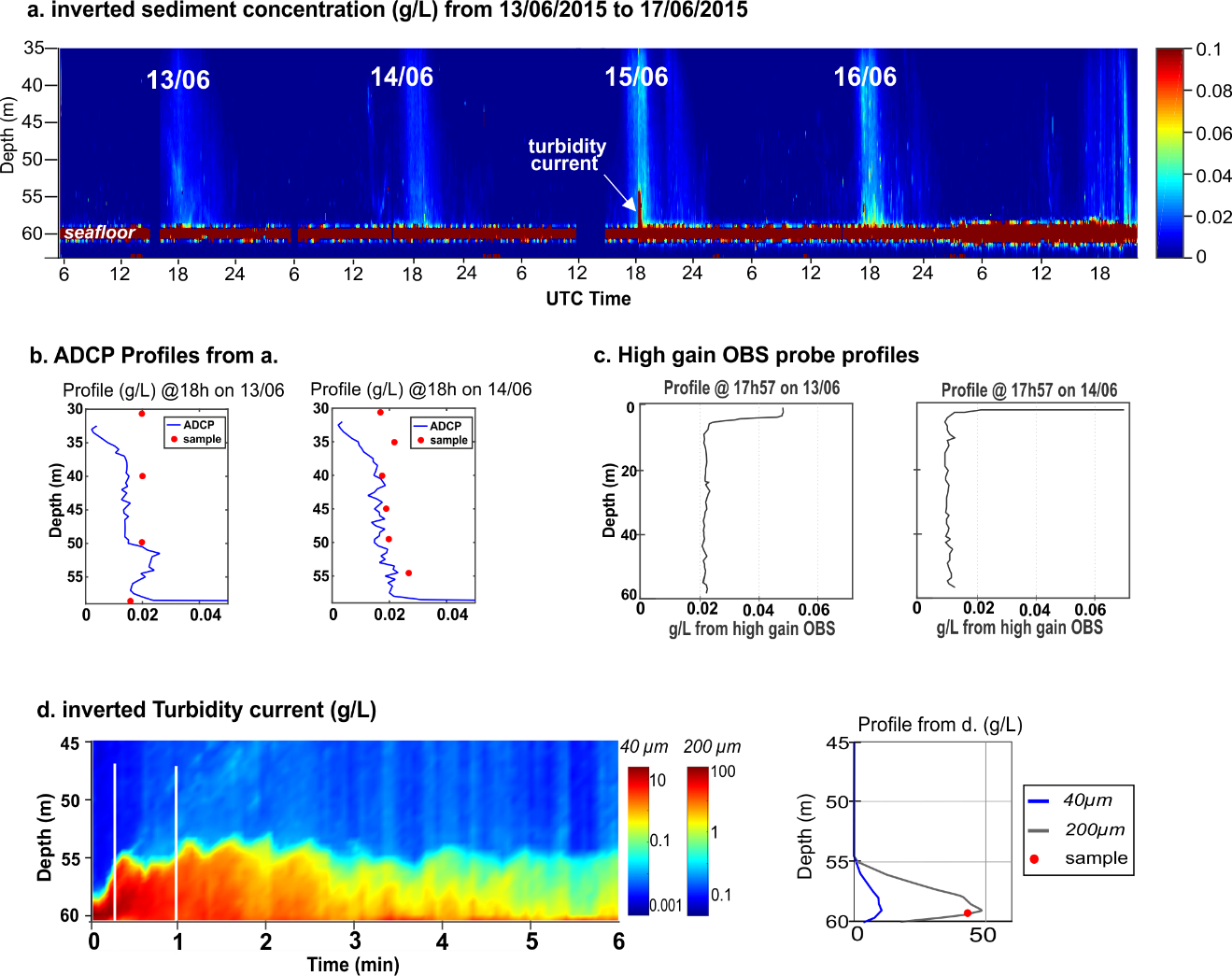
Figure S7. Acoustic Doppler Current Profiler inversion results (see Fig. 1b for ADCP location). a. sediment concentration for 5 days of measurements (mean grain size used for inversion = 40μ). b. profiles from a. plotted against sediment samples taken at same time/location. c. suspended sediment (g/L) after calibration of high gain probe. d. zoom into turbidity current sediment concentration (D50 grain size used for inversion = 40 and 200μ).


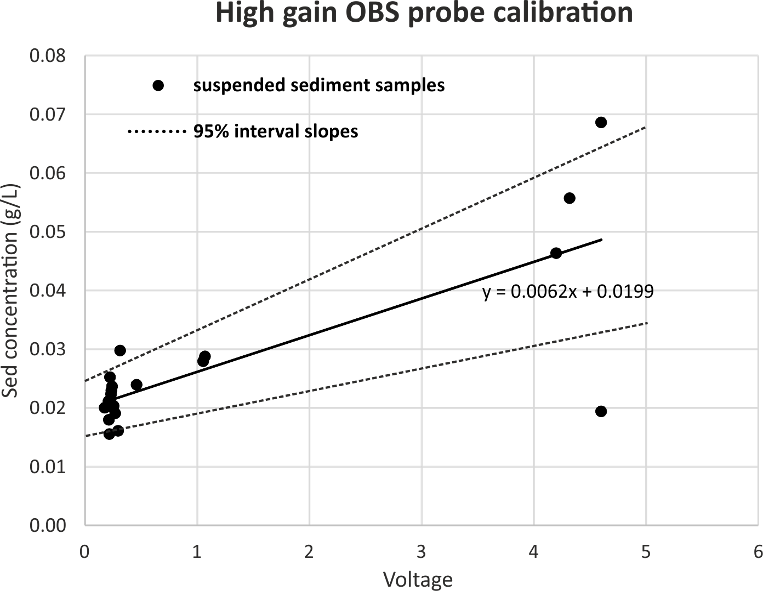


**Figure S8.** Calibration curve for the high gain Optical Backscatter probe deployed from the Moving vessel (Fig. 1c). Dots are physical samples collected in the water column at the same time as the OBS deployment


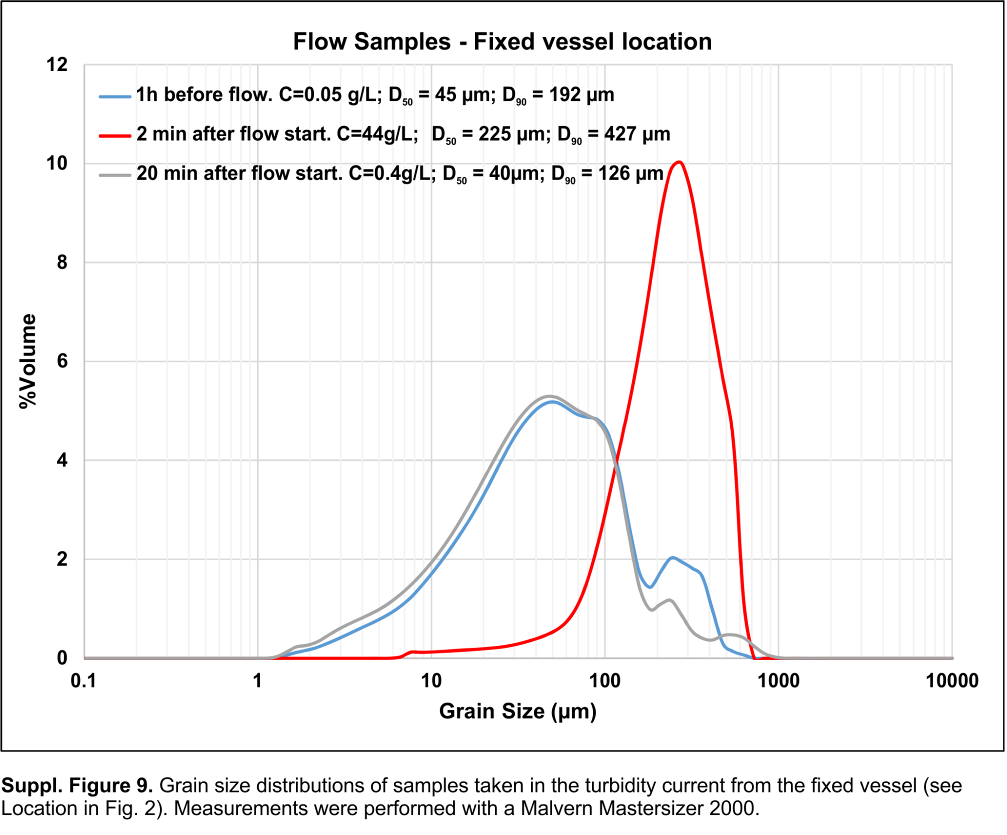


**Figure S9.** Grain size distributions for samples taken in the turbidity current from the fixed vessel (Location in Fig. 2). Measurements were performed using a Malvern Mastersizer 2000.


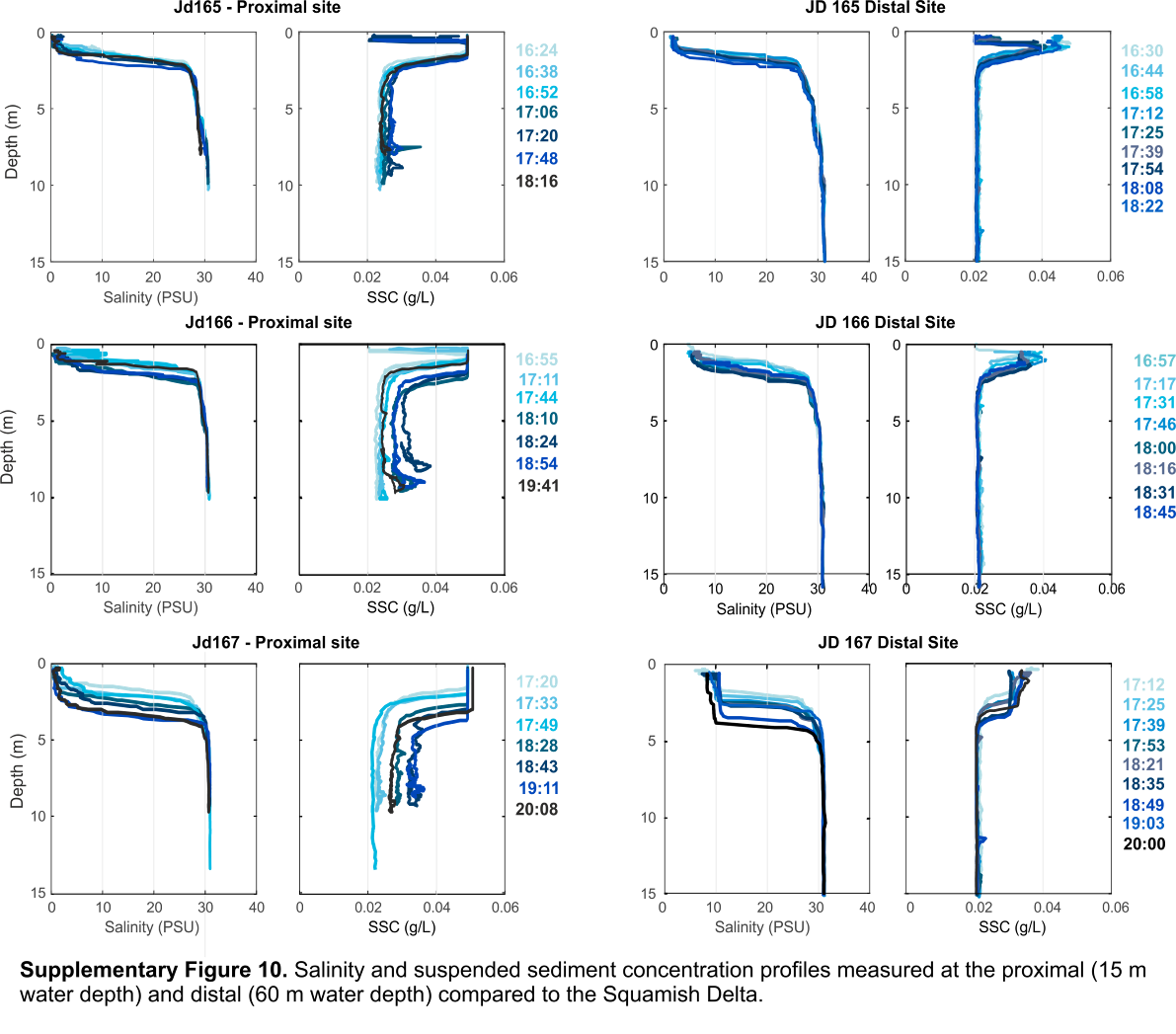


**Figure S10.** Salinity and suspended sediment concentration profiles measured at the proximal (15 m water depth) and distal (60 m water depth) compared to the Squamish delta (see Fig. 4 for location). Note: the abrupt cut-off at 0.05 g/L at the proximal site indicates saturation of the instrument. The maximum suspended sediment values are further constrained by direct samples collected in the river shown in Table S11.


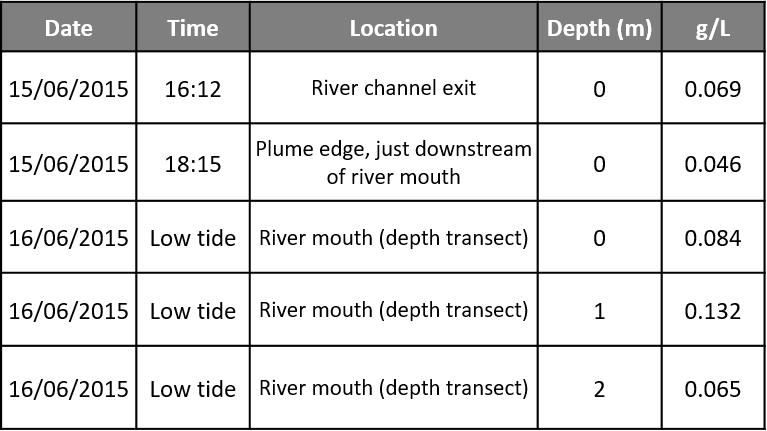


**Table S1.** Water and sediment samples collected in the Squamish river mouth.


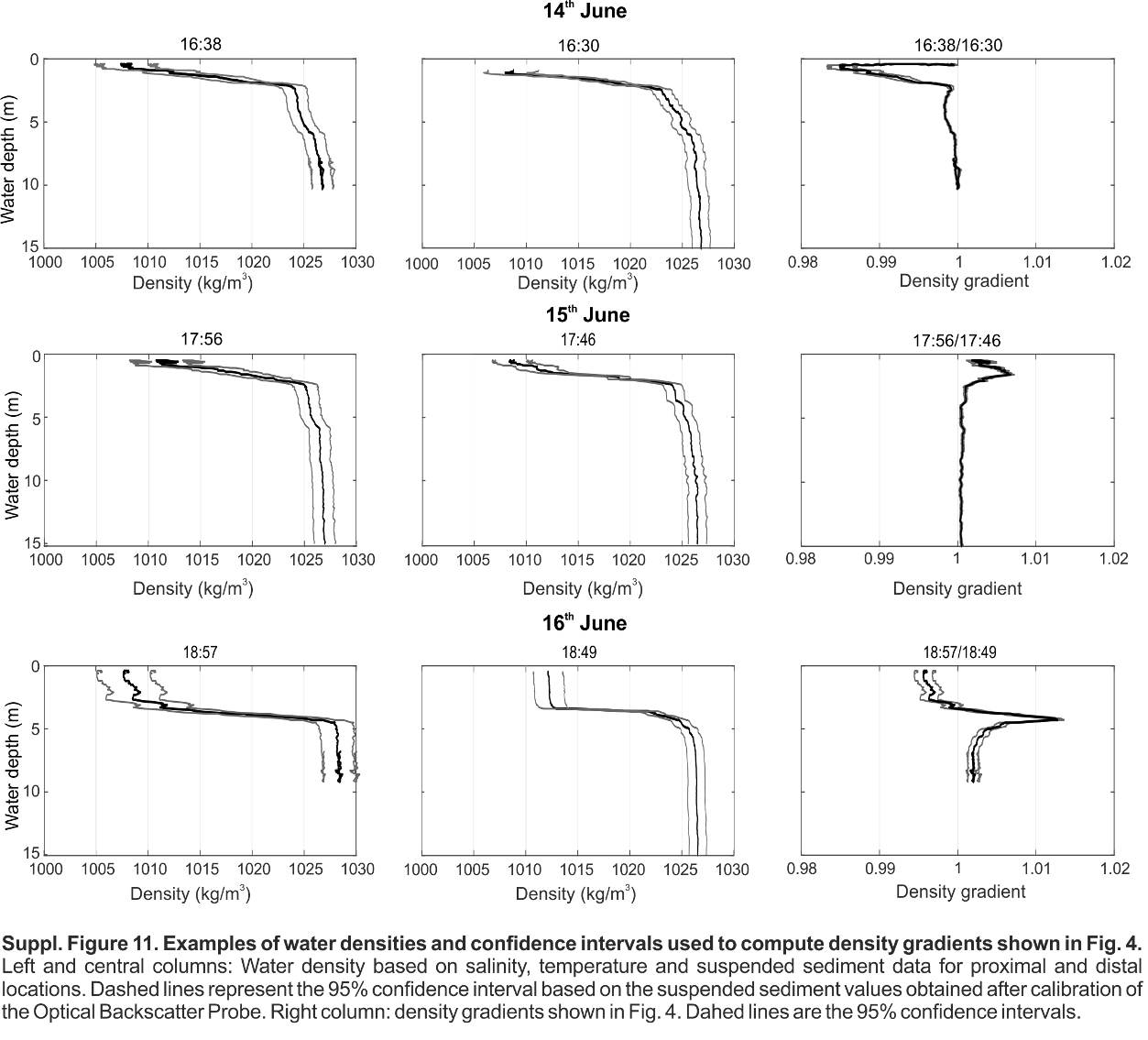


**Figure S12.** Examples of water densities and confidence intervals used to compute density gradients shown in Fig. 4. Left and central columns: water density based on salinity, temperature and suspended sediment data for proximal and distal locations. Dashed lines represent the 95% confidence intervals based on the suspended sediment values obtained after calibration of the Optical Backscatter probe. Right column: Density gradients shown in Fig. 4. Dashed lines are the 95% confidence intervals.


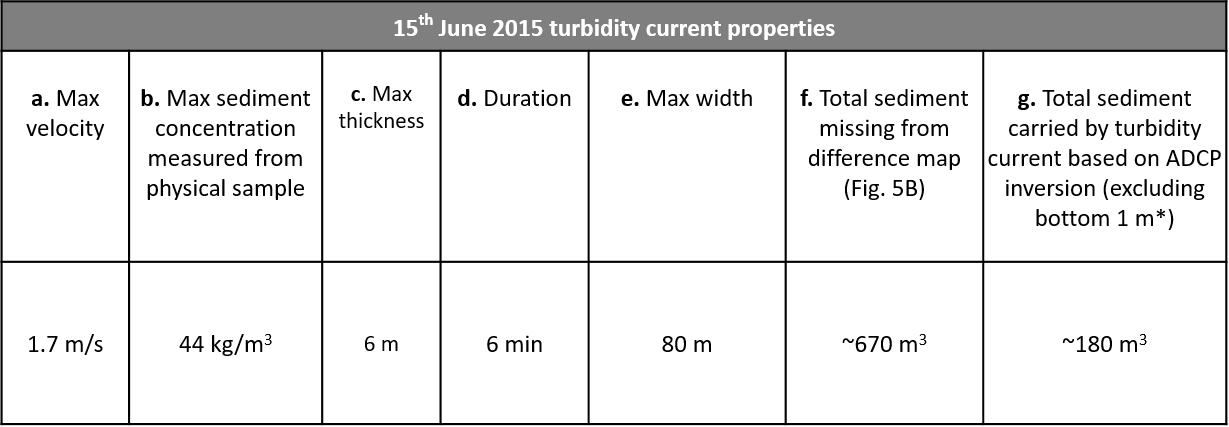


**Table S2.** Properties of the turbidity current monitored on 15^th^ June 2015 and presented in this study. **a.** Maximum velocity of the flow front imaged by a forward-looking echosounder as presented in Hage et al. (2018). **b.** Maximum sediment concentration measured from a sample taken 2 min after the flow started (Fig. S9). **c.** Maximum thickness documented using acoustic Doppler current profiler backscatter (Fig. 3F). **d.** Turbidity current duration measured by the acoustic Doppler current profiler (Fig. 3G). **e.** Maximum width of the flow imaged by a forward-looking echosounder, as presented in Hage et al. (2018). Flow remained confined within the 90 m long submarine channel. **f.** Total sediment volume lost from the seabed, based on a comparison between two bathymetric surveys collected before and after the turbidity current, Fig. 5B). * Note that this method does not include thin deposits below the resolution of bathymetric mapping, which may reduce this estimate of total sediment volume lost. **g.** Total sediment volume carried by the turbidity current, based on inversion of backscatter data from the acoustic Doppler current profiler. * Note that the ADCP did not image the bottom meter of the flow, due to acoustic interference with the seabed, and this method may thus underestimate sediment volumes transported by the flow.
